# Cyclin dependent kinase 14 as a paclitaxel-resistant marker regulated by the TGF-β signaling pathway in human ovarian cancer

**DOI:** 10.7150/jca.86842

**Published:** 2023-08-15

**Authors:** Wencai Guan, Jia Yuan, Xin Li, Xuzhu Gao, Fanchen Wang, Huiqiang Liu, Jimin Shi, Guoxiong Xu

**Affiliations:** 1 Research Center for Clinical Medicine, Jinshan Hospital, Fudan University, Shanghai 201508, China;; 2 Department of Oncology, Shanghai Medical College, Fudan University, Shanghai 200032, China.

**Keywords:** CDK14, chemoresistance, ovarian cancer, reversal, TGF-β signal

## Abstract

Cyclin dependent kinase 14 (CDK14) plays a central role in the control of cell proliferation and cell cycle progression. However, the specific function and regulatory mechanism of CDK14 on paclitaxel (PTX) resistance in ovarian cancer (OC) remain unclear. The present study demonstrated that CDK14 was overexpressed in OC tissues and cells at mRNA and protein levels detected by qRT-PCR, Western blot, and immunohistochemistry. Survival analysis showed that elevated CDK14 was related to the poor prognosis of OC patients. Overexpression of CDK14 was correlated with chemoresistance in OC. The expression level of CDK14 was higher in PTX-resistant OC cells (SK3R-PTX and OV3R-PTX) than in their counterpart-sensitive cells (SK-OV-3 and OVCAR-3). Knockdown of CDK14 decreased multidrug resistance 1 (MDR1) and β-catenin expression in SK3R-PTX and OV3R-PTX cells and resensitized OC cells to PTX by decreasing cell proliferation and inducing cell apoptosis. Administration of transforming growth factor (TGF)-β1 decreased CDK14 protein in PTX-resistant OC cells. The inhibitory effect of TGF-β1 on CDK14 expression was abolished in the presence of a TGF-β type I receptor kinase inhibitor (SB-431542). Furthermore, TGF-β signal transducer Smad2 protein directly bound to the region -437 to -446 upstream of the CDK14 transcription start site (TSS), resulting in downregulating the expression of CDK14. These data indicate that CDK14 is a PTX-resistant marker and is regulated by the TGF-β signaling pathway. Targeting CDK14 to enhance the sensitivity of PTX may provide a new therapeutic strategy for reversing the PTX resistance in OC.

## Introduction

Ovarian cancer (OC) is the fifth leading cause of cancer-related death in women worldwide 1. About 19,880 newly diagnosed cases and 12,810 deaths of OC patients are estimated in 2022 in the United States 2. The reduction of OC mortality has not been improved significantly for decades due to the lack of early diagnostic markers at the early stage and effective treatment at the advanced stage of the disease. About 75% of OC patients are diagnosed at stages III-VI when they first visit a hospital 3, 4. The initial treatment for advanced OC is debulking surgery and adjuvant therapy, including chemotherapy with cures promising up to 50% 5, 6. Paclitaxel (PTX) is one of the first-line chemotherapeutic agents for an advanced disease but some patients eventually develop chemoresistance 7, 8. The underlying molecular mechanisms of PTX resistance in OC remain unclear. It has been shown that multidrug resistance 1 (MDR1, also termed ABCB1 and P-gp) and Wnt signaling are involved in chemoresistance and cell proliferation 9, 10.

Cyclin dependent kinase 14 (CDK14, also known as PFTK1 and PFTAIRE1) is a serine/threonine kinase that plays a central role in the control of cell proliferation and cell cycle progression 11. Human CDK14 expression is relatively high in the brain, heart, kidney, ovary, pancreas, and testis 12. Overexpressed CDK14 has been reported in many cancers, including breast 13, gastric 14, non-small cell lung 15, and ovarian 16 cancers. Furthermore, a high level of CDK14 predicts a poor prognosis and resistance to chemotherapy in esophageal squamous cell carcinoma 17. However, the specific function and regulatory mechanism of CDK14 on PTX resistance in OC remain unknown.

Transforming growth factor-β (TGF-β) signaling plays an essential role in cellular processes involved in cell growth, differentiation, and death. The dysregulation of the TGF-β signaling pathway causes many diseases such as cancer 18. In the early stage of cancer, TGF- β exhibits anti-tumor functions, including cell cycle arrest and apoptosis induction, whereas in the late stage of cancer, it promotes tumorigenesis, including metastasis and chemoresistance 19. Previous studies have shown that TGF-β affects chemoresistance in a variety of solid tumors 20, 21. Cellular mechanisms of chemoresistance include impaired apoptosis, enhanced DNA repair, drug influx, autophagy, epithelial-mesenchymal transition (EMT), and cancer stemness 22, 23. It has been shown that TGF-β signaling promotes EMT and induces stem-like properties that contributed to chemoresistance in OC 24, 25.

Here we examined the expression and characteristics of CDK14 in OC and PTX-resistant cells and the prognosis value in OC patients. The influence of CDK14 on drug transporter MDR1 and its regulator β-catenin was also evaluated. Finally, TGF-β signaling-regulated CDK14 expression was explored. The mechanism underlying TGF-β-mediated CDK14- regulated MDR1 expression via β-catenin may contribute to the reversal of PTX resistance.

## Materials and methods

### Cell lines and culture

All cells used in the study were derived from human ovaries. Non-tumorous IOSE-80 cells (originally derived from normal ovarian epithelium) (ZSGB-BIO, Beijing, China) were cultured in the RPMI-1640 medium (Gibco, Invitrogen, Carlsbad, CA, USA) with 10% fetal bovine serum (FBS, Biological Industries, Kibbutz Beit-Haemek, Israel). OC cell line OVCAR-3 (originally derived from ascites of a high-grade serous ovarian adenocarcinoma) (American Type Culture Collection, ATCC, Manassas, VA, USA) and OV3R-PTX (generated by this laboratory) 26 were cultured in RPMI-1640 medium (Gibco) with 20% FBS. OC cell line SK-OV-3 (originally derived from ascites of an ovarian endometrioid adenocarcinoma) (ATCC) and SK3R-PTX (generated by this laboratory) 26 were cultured in McCoy's 5A (Biological Industries) medium with 10% FBS. All cells were in a humidified incubator at 37 ℃ with 5% CO2. Cell lines were authenticated by short tandem repeat (STR) analysis with routine detection of pathogenic-free and mycoplasma negative.

### Transforming growth factor-beta (TGF-β) treatment

PTX-resistant OC cells were treated with TGF-β1 (R&D Systems, Minneapolis, MN, USA) at different doses (10 or 20 ng/mL) as indicated for 48 h. For blocking the inhibitory effect of TGF-β1 on CDK14 expression, cells were pretreated with a TGF-β type I receptor kinase inhibitor SB-431542 (10 mM, Sigma, Saint Louis, MO, USA) 27 for 0.5 h prior to TGF-β1 treatments.

### Cell transfection

The X-tremeGENE small interfering RNA (siRNA) Transfection Reagent (Roche Applied Science, Indianapolis, IN, USA) was used for the transfection of CDK14-siRNAs (si-CDK14) or negative control-siRNA (si-NC) and plasmids according to the manufacturer's instructions. The siRNAs for CDK14 were synthesized by Shanghai GenePharma Co., Ltd. The sequences of siRNA are listed in Supplementary Table S1.

### Plasmid vector construction and transfection

Short hairpin RNAs (shRNAs) targeting CDK14 mRNA (sh-CDK14) and negative control-shRNA (sh-NC) were inserted into the knockdown plasmid (Genewiz, Suzhou, China). The sequences of shRNA are listed in Supplementary Table S1. These knockdown plasmids were co-transfected with PSPAX2 and PMD2G plasmids to generate sh-RNA lentiviruses in HEK293T cells (FuHeng Biology, Shanghai, China) by using lipoD293 transfection reagent (SignaGene Laboratories, Frederick, MD, USA) following the manufacturer's protocol. Lentiviral supernatant was collected at 48 and 72 h after transfection. After SK3R-PTX and OV3R-PTX cells reached 20-30% confluence in 12-well plates, the lentiviral supernatant mixed with the complete medium was added in the presence of 10 μg/mL Polybrene (HANBIO, Shanghai, China).

### RNA extraction and quantitative real-time PCR (qRT-PCR)

Total RNA was extracted using an RNA-Quick Purification Kit (ES Science, Shanghai, China). PCR was applied using a qPCR RT kit (Mei5 Biotechnology, Beijing, China). The threshold cycle (Ct) was determined using the 7300 real-time PCR system (V1.4, Applied Biosystems, USA). The condition for PCR amplification was initial denaturation at 95 ℃ for 1 min followed by 40 cycles of denaturation at 95 ℃ for 10 sec and annealing/elongation at 60 ℃ for 30 sec. β-actin was used as an internal control for gene expression. The PCR primer sequences are listed in Supplementary Table S1.

### Protein extraction and Western blot analysis

The total protein was extracted from cells lysed with sodium dodecyl sulfate (SDS, Beyotime Biotechnology, Shanghai, China) lysate containing 1% phenylmethanesulfonyl fluoride (Beyotime Biotechnology) and 1% phosphatase inhibitor. The cytoplasmic and nuclear proteins were isolated with the Minute™ Cytoplasmic & Nuclear Extraction Kit (#SC-003; Invent Biotechnologies, Inc, Minnesota, USA) according to the manufacturer's instructions. The protein concentration was measured with the BCA protein assay kit (Beyotime Biotechnology). Protein samples were run on SDS-polyacrylamide gel electrophoresis (SDS- PAGE) and transferred to PVDF membranes. After blocking with 5% nonfat milk for 1 h at room temperature, the membrane was incubated with the primary antibody at 4 ℃ overnight. The following antibodies were used in the study: anti-CDK14 antibody (1:1000 dilution, Santa Cruz Biotechnology, Inc, Dallas, Texas USA), anti-MDR1 (1:5000 dilution, P-gp, Cell Signaling Technology, Inc., Boston, MA, USA), and anti-β-actin (1:5000 dilution, Proteintech, Wuhan, China). The secondary antibodies of the anti-mouse IgG or anti-rabbit IgG were used at RT for 1 h. The protein bands were photographed by the chemiluminescence imaging system (Tanon Science & Technology, Shanghai, China).

### Immunohistochemistry (IHC) staining

A total of 18 paraffin-embedded ovarian cancer tissues derived from OC patients and 8 non-tumorous ovarian tissues derived from non-cancerous patients with benign cytes were obtained from Jinshan Hospital, Fudan University. The inclusion criteria for OC patients were (1) Female; (2) Age 18-75 years; (3) Pathological confirmation of epithelial OC after operation; (4) Neither received chemotherapy nor radiotherapy; (5) Archived tumor tissue samples within 5 years in Jinshan Hospital. The exclusive criteria were (1) Incomplete specimens of patients; (2) Not primary OC tissues; (3) Incomplete clinical data. Ethics approval was approved by the Ethics Committee of Jinshan Hospital (No. JYLLKY-2019-01-01). IHC analysis was performed and signal scores were measured as described previously 27. Briefly, the CDK14 score was represented by the staining index (SI) which was calculated by the sum of (a) one of the staining intensity scores: 0 (-), 1 (+), 2 (++), 3 (+++) and (b) one of the percentage scores of immuno-positive cells: 0 (no positive cells), 1 (≤ 25%) 2, (26-50%), 3 (51-75%), 4 (> 75%). Then, patients were divided into two groups: low expression (0-4 sum points) and high expression (5-7 sum points) based on the SI of CDK14.

### Immunofluorescent imaging

Cells were seeded in a 35-mm confocal culture dish with a 20-mm glass bottom at a volume of 0.5 mL/dish. After confluence reached 50-70%, cells were fixed with 4% paraformaldehyde (PFA) for 15 min and then washed with phosphate-buffered saline (PBS) for 5 min once. After cells were permeabilized with 0.1% Triton X-100 in PBS for 15 min, a 0.5 mL solution of QuickBlock™ Blocking Buffer for Immunol Staining (Beyotime Biotechnology) was added to cells and incubated for 1 h at RT. After cells were incubated with the primary antibody anti-CDK14 at 4 ℃ overnight, the secondary antibody (ZSGB-BIO, Beijing, China) was incubated for 1 h at RT in a dark place. Further stained with DAPI (Beyotime Biotechnology) for 5 min and washed with PBS 2 times, immunofluorescent images were taken by a BioTek Cytation C10 Confocal Image Reader (Agilent Technologies, Beijing, China).

### PTX cytotoxicity assay and IC_50_ measurement

shRNA-infected SK3R-PTX or OV3R-PTX cells were seeded in 96-well plates at a density of 7×10^3^ cells/well. After treatment with different concentrations of PTX for 48h, the PTX toxicity in the cells was determined using a CCK-8 kit for cell viability measurement. Cell resistance to PTX was determined by the measurement of a half-maximal inhibitory concentration (IC50). Next, an IC50 dose of PTX was applied to the cells and the cell viability was detected by the CCK-8 kit after 24, 48, and 72 h treatment.

### Flow cytometry of cell cycle and apoptosis

For cell cycle detection, the detailed method was described previously 27. After plating overnight, PTX-resistant SK3R-PTX and OV3R-PTX cells were transiently transfected with si-CDK14 or si-NC for 48 h. The cell cycle was then measured by flow cytometry (Gallios, Beckman Coulter, Inc., Brea, CA, USA). For apoptotic cell detection, sh-CDK14 or sh-NC stably-expressed SK3R-PTX and OV3R-PTX cells were cultured in 6-well plates and treated with or without PTX for 48 h. After detaching cells with an EDTA-free trypsin (GENOM BIO, Hangzhou, Zhejiang), cell suspension at a density of 1×10^6^/100 μL was transferred into a 5 mL tube, followed by adding 1 μL of Annexin-V-FITC and/or 3 μL of propidium iodide (PI) according to the product instructions (BD Biosciences, San Jose, CA, USA). After incubation with 400 μL of 1×binding buffer in the dark for 15 min, apoptotic cells were detected by flow cytometry (Gallios, Beckman Coulter, Inc., Brea, CA, USA).

### Dual-luciferase reporter assay

To validate Smad2 binding to CDK14 promoter directly, three different lengths of CDK14 promoter with or without Smad2 binding site were amplified and ligated into the pGL4-Basic vector (Promega, USA). SK3R-PTX and OV3R-PTX cells were co-transfected with the control Renilla luciferase vector pRL-SV40 for 24 h. After treatment with 20 ng/mL TGF-β1 for 48 h, the luciferase activity was measured.

### Analysis of chemotherapeutic responsiveness

RNA-sequencing expression profiles (level 3) and their corresponding clinical information for serous cystadenocarcinoma (OV) were downloaded from the TCGA dataset (https://portal.gdc.com). Information on predicting the chemotherapeutic responsiveness for each sample was obtained from the largest publicly available pharmacogenomics database, the Genomics of Drug Sensitivity in Cancer (GDSC) (https://www.cancerrxgene.org/). The prediction process was implemented by the R package “pRRophetic”. All parameters were set as the default values. Using the batch effect of combat and the type of all tissues, the duplicate gene expression was summarized as the mean value.

### Prognostic value analysis

The prognostic value of CDK14 was assessed by the online database, Kaplan-Meier Plotter (www.kmplot.com) 28. Survival data in OC patients with CDK14 expression were obtained using PrognoScan (http://dna00.bio.kyutech.ac.jp/PrognoScan/) 29 and analyzed by GEO.

### Gene set enrichment analysis (GSEA)

Gene expression RNA-seq data (n = 10286 in total) across 33 TCGA cancer types were collected from the Xena TCGA hub (https://xena.ucsc.edu/). To determine the effects of CDK14 in cancers, each type of cancer was divided into CDK14-high and CDK14-low groups. GSEA analysis was applied to every cancer type and was performed using GSEA software (Ver 4.2.2). The hallmark gene sets (Ver 7.5.1) were used for functional annotations. Normalized p- value < 0.05 and false discovery rate q-value < 0.25 were set as cut-offs to define the significance of enriched hallmarks.

### Cancer Cell Line Encyclopedia (CCLE)

The CCLE database (https://sites.broadinstitute.org/ccle) contains in-depth analyses of multi-omics maps of thousands of cancer cell lines and information on genetic mutations, RNA splicing, DNA methylation, and histone modification in more than 1,000 cell lines. The RNA- seq data in OC cell lines were processed. Genes significantly correlated with CDK14 expression were screened out. The enrichment analyses were conducted by KEGG and GO by R packages. The GSEA was also employed to dissect the signaling pathways correlated with CDK14 expression (CDK14^high^ *vs.* CDK14^low^).

### Statistical analysis

All data were analyzed through GraphPad Prism 8.0 (GraphPad Software Inc.) and R version 4.1.3 (R Foundation for Statistical Computing, Vienna, Austria). A student's *t*-test was used in a two-group comparison. One-way ANOVA followed by a Tukey's test was used to perform a comparison for continuous variables among groups ≥3. For a non-parametric analysis, a Fisher's exact test or a Wilcoxon test was used according to the experiments. A P-value < 0.05 was considered as statistical significance.

## Results

### CDK14 is overexpressed in OC and relates to the poor prognosis

**Figure 1:**
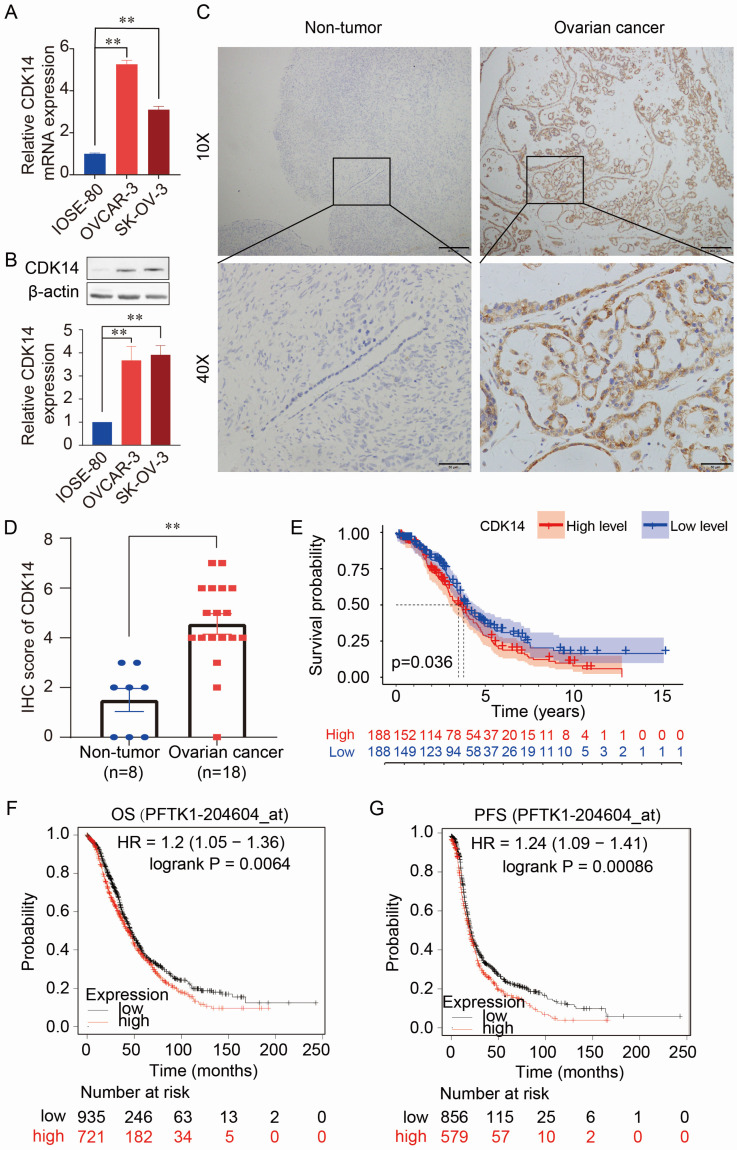
Expression and prognostic value of CDK14 in ovarian cancer. (A) Detection of CDK14 mRNA expression in a non-tumorous immortalized ovarian epithelial cell line (IOSE- 80) and epithelial ovarian cancer cell lines (OVCAR-3, SK-OV-3) by qRT-PCR. (B) Detection of CDK14 protein expression in IOSE-80, OVCAR-3, and SK-OV-3 cells by Western blot analysis. Histogram shows the semi-quantification analysis of blotting bands by ImageJ. (C) Detection of CDK14 protein in non-tumorous tissue and ovarian cancer tissue by immunohistochemistry (IHC) staining. Representative images are shown. Original magnification ×100, scale bar 200 μm; partial magnification ×400, scale bar 50 μm. (D) Histogram shows the comparison of CDK14 protein expression between non-tumorous ovarian tissues (n=8) and primary ovarian cancer tissues (n=18) after IHC staining. (E) Association of overall survival (OS) with high- or low-expression of CDK14 was analyzed through TCGA- OV data. (F, G) OS and progression-free survival (PFS) were analyzed through mRNA gene chip data by Kaplan-Meier Plotter. **, P < 0.01.

The expression levels of CDK14 mRNA and protein were higher in OC cells (OVCAR-3 and SK-OV-3) than in normal human immortalized ovarian surface epithelial cells (IOSE-80) detected by qRT-PCR (Fig. 1A) and Western blot (Fig. 1B). Immunohistochemistry (IHC) staining also showed higher expression of CDK14 in the OC tissue (n=18) than in the non-tumorous ovarian tissues (n=8) (Fig. 1C and 1D). Further analysis showed that high expression of CDK14 was positively correlated with the high tumor stage (P = 0.026) (Supplementary Table S2). Furthermore, the high level of CDK14 expression was correlated with the shorter survival time of patients by analyzing TCGA-OV data (Fig. 1E). In addition, a survival analysis from mRNA gene chip data by Kaplan-Meier Plotter also showed a negative correlation between CDK14 expression and overall survival (OS)/progression-free survival (PFS) in OC (Fig. 1F and 1G). Further analyses of the prognostic value from GSE datasets (GSE9891 and GSE26712) showed that OC patients with a higher expression level of CDK14 mRNA had unfavorable OS and disease-free survival (DFS) (Fig. S1A-S1C).

### High expression of CDK14 is correlated with chemoresistance in OC

Next, we processed the RNA-seq data in ovarian cell lines downloaded from the Cancer Cell Line Encyclopedia (CCLE) database (https://sites.broadinstitute.org/ccle) to search CDK14-correlated genes. We found that there were 87 genes significantly associated with CDK14 expression by correlation test (P < 0.001) in the RNA-seq data from 47 OC cell lines. KEGG analysis showed that CDK14-correlated (positively or negatively) genes gathered at the pathways of chemoresistance, transcriptional misregulation in cancer, etc (Fig. S2A). GO term analyses, including biological process (BP), cellular component (CC), and molecular function (MF), showed that these genes may involve in the apoptotic process, response to a toxic substance, etc (Fig. S2B).

**Figure 2:**
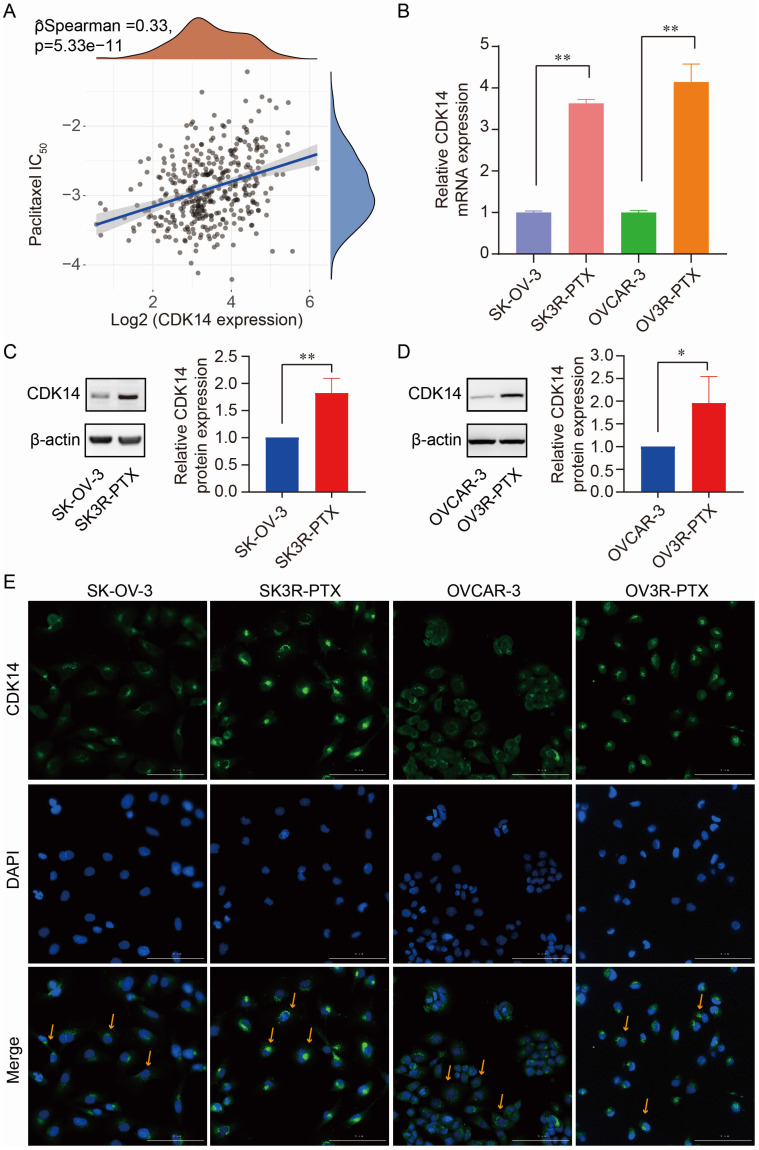
Correlation of CDK14 expression with PTX resistance. **(A)** Spearman correlation analysis of IC50 score of PTX with CDK14 expression in OC patients (n=376) from the TCGA database. The abscissa represents gene expression distribution, the ordinate represents the IC50 score distribution, and the density curve on the right represents the IC50 score distribution trend. **(B)** Detection of CDK14 mRNA expression in PTX-sensitive (SK-OV-3, OVCAR-3) and PTX-resistant (SK3R-PTX, OV3R-PTX) cancer cells by qRT-PCR. (C, D) Detection of CDK14 protein expression in SK-OV-3, SK3R-PTX, OVCAR-3, and OV3R-PTX cells by Western blot. Histograms show the semi-quantification analysis of blotting bands by ImageJ. (E) Immunofluorescence staining of CDK14 in SK-OV-3, SK3R-PTX, OVCAR-3, and OV3R- PTX cells. Arrows point to the representative positive cells. Magnification ×200; scale bar, 200 μm. *, P < 0.05; **, P < 0.01.

The correlation of CDK14 expression with PTX resistance was detected by Spearman correlation analysis of IC50 score with CDK14 expression in OC patients (n = 376) data extracted from the TCGA-OV database. We found that IC50 scores of PTX positively correlated with the expression of CDK14 (Fig. 2A), indicating that cells with high CDK14 expression were more resistant to PTX. Indeed, the expression level of CDK14 mRNA was higher in PTX-resistant cells (SK3R-PTX and OV3R-PTX) than in PTX-sensitive cells (SK-OV-3 and OVCAR-3) detected by qRT-PCR (Fig. 2B). Western blot analysis confirmed high expression of CDK14 protein in SK3R-PTX and OV3R-PTX cells compared with SK- OV-3 and OVCAR-3 cells (Fig. 2C and 2D). Furthermore, immunofluorescence (IF) staining of CDK14 showed more signals in SK3R-PTX and OV3R-PTX cells than in SK-OV-3 and OVCAR-3 cells, and these bright IF signals of CDK14 were mainly localized in the cytoplasm (Fig. 2E).

### Knockdown of CDK14 decreases MDR1 and β-catenin

**Figure 3:**
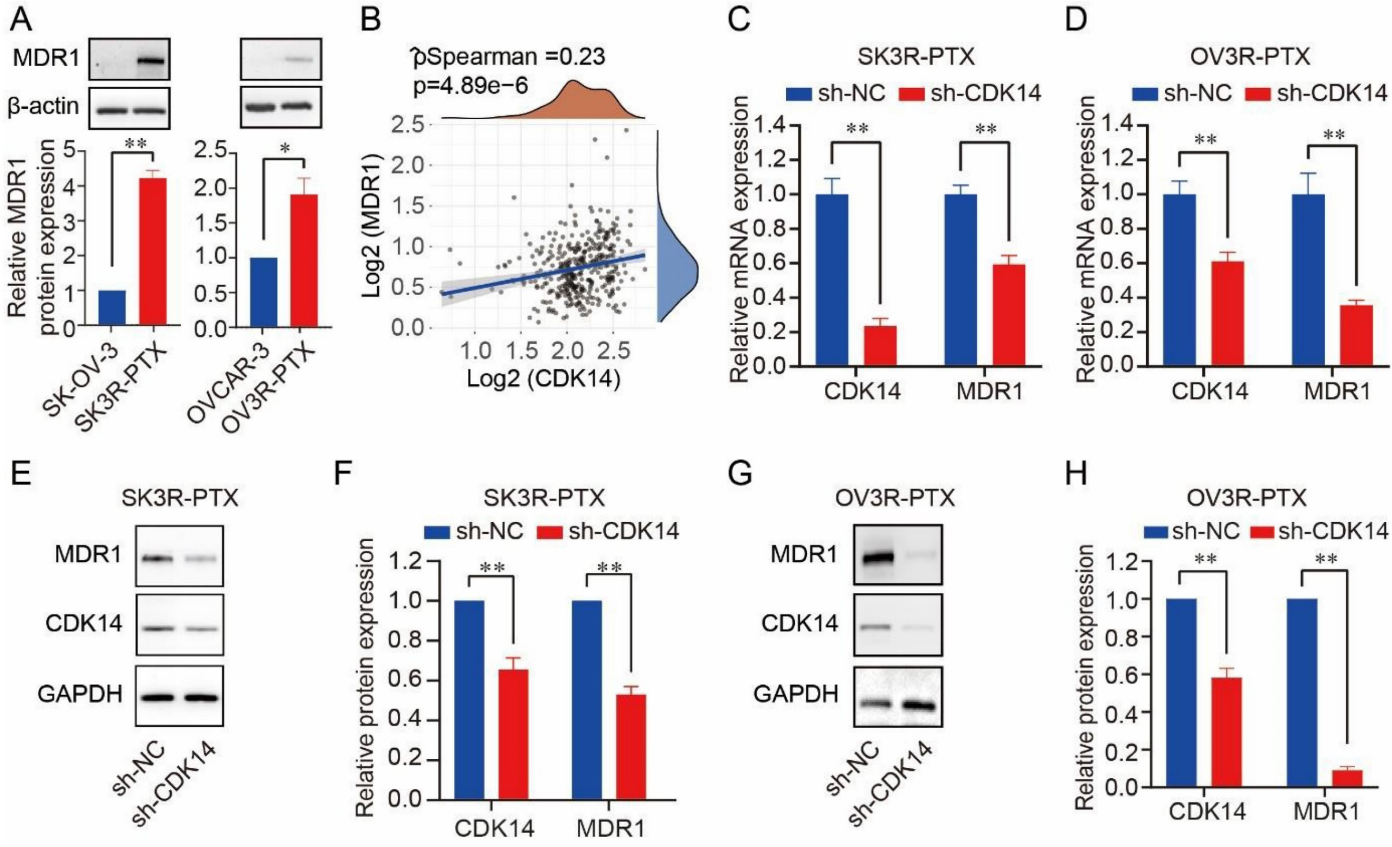
Correlation of CDK14 expression with MDR1 expression. (A) Expression of MDR1 protein in PTX-resistant OC cells (SK3R-PTX and OV3R-PTX) and their parental PTX-sensitive OC cells (SK-OV-3 and OVCAR-3) detected by Western blot. Histograms show the semi-quantification analysis of blotting bands by ImageJ. (B) Spearman correlation analysis of CDK14 and MDR1 expression in OC patients (n=376) from the TCGA database. The abscissa represents CDK14 expression distribution; the ordinate represents MDR1 expression distribution; the density curve on the right represents the MDR1 expression distribution trend. (C, D) Detection of CDK14 and MDR1 mRNA expression in SK3R-PTX and OV3R-PTX cells by qRT-PCR after cells transfected with sh-NC and sh-CDK14. (E-H) Detection of the MDR1 and CDK14 protein expression in SK3R-PTX and OV3R-PTX cells by Western blot after cells transfected with sh-NC and sh-CDK14. Histograms show the semi-quantification analysis of blotting bands by ImageJ. n = 3; *, P < 0.05; **, P < 0.01.

A high level of MDR1 was found in SK3R-PTX and OV3R-PTX cells compared to SK- OV-3 and OVCAR-3 (Fig. 3A). Since both CDK14 and MDR1 were overexpressed in two PTX-resistant cells, we next analyzed the relationship between CDK14 and MDR1. A positive correlation of CDK14 mRNA with MDR1 mRNA expression was found in 376 OC patients in the TCGA-OV database (Fig. 3B). After CDK14 knockdown in PTX-resistant OC cells, a decrease in MDR1 mRNA and protein was observed by qRT-PCR (Fig. 3C and D) and Western blot (Fig. 3E-3H), respectively, in SK3R-PTX and OV3R-PTX cells.

It has been reported that MDR1 expression is regulated by the Wnt/β-catenin signaling pathway. Next, we examined whether CDK14 regulates MDR1 in PTX-resistant cells via β- catenin. We found that the CDK14 protein was detectable in the cytoplasm rather than in the nucleus in SK3R-PTX and OV3R-PTX cells after infection with the CDK14-shRNA virus (Fig. S3). Knockdown of CDK14 decreased cytoplasmic β-catenin protein in SK3R-PTX cells and nuclear β-catenin protein in SK3R-PTX and OV3R-PTX cells, suggesting that CDK14- regulated MDR1 expression is most likely via the Wnt/β-catenin signaling pathway.

### Knockdown of CDK14 sensitizes OC cells to PTX

**Figure 4:**
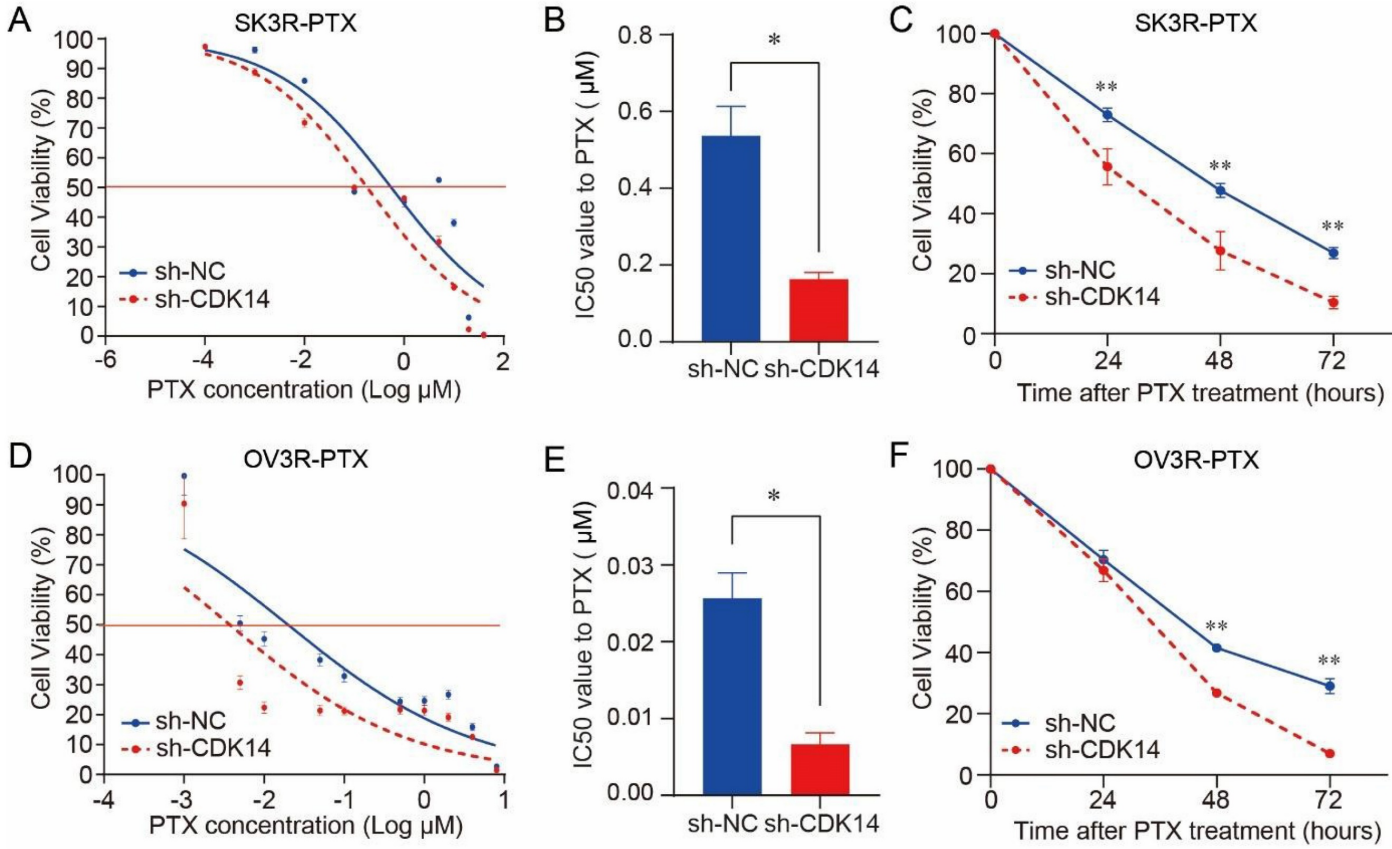
Effect of CDK14 on paclitaxel sensitivity. Cell viability was detected by the CCK-8 assays. (A) Detection of SK3R-PTX cell viability after CDK14 knockdown in the presence of different doses of PTX for 48 hours. (B) Measurement of PTX IC50 value from A. (C) Detection of SK3R-PTX cell viability after CDK14 knockdown in the presence of IC50 dose of PTX from B for different time points. (D) Detection of OV3R-PTX cell viability after CDK14 knockdown in the presence of different doses of PTX for 48 hours. (E) Measurement of PTX IC50 value from D. (F) Detection of OV3R-PTX cell viability after CDK14 knockdown in the presence of IC50 dose of PTX from E for different time points. *, P < 0.05; **, P < 0.01.

PTX cytotoxicity assay showed that the knockdown of CDK14 effectively increased the sensitivity of SK3R-PTX cells to PTX in a dose-dependent manner (Fig. 4A) and the IC50 value was decreased in SK3R-PTX cells (Fig. 4B). Time-course study showed that the cell viability was decreased in sh-CDK14 infected SK3R-PTX cells after 24, 48, and 72 h treatment of PTX using an IC50 dose (Fig. 4C). The same phenomenon was also observed in OV3R-PTX cells after CDK14 knockdown (Fig. 4D-4F). These data indicate that the reduction of CDK14 may reduce the resistance or enhance the sensitivity of PTX-resistant cells to PTX.

### Knockdown of CDK14 decreases PTX-resistant OC cell proliferation and induces PTX-resistant OC cell apoptosis

**Figure 5:**
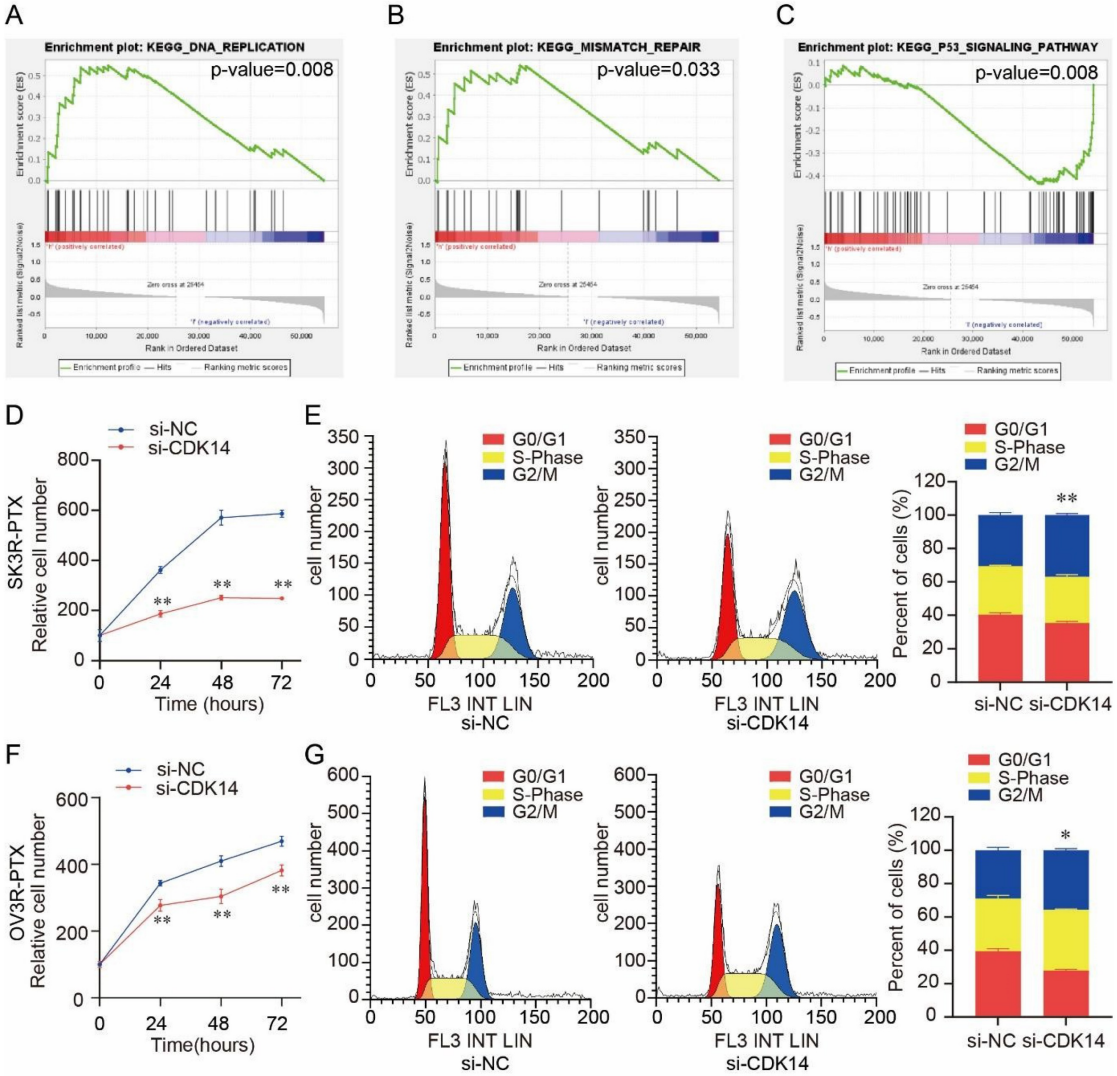
Effect of CDK14 on cell proliferation. (A-C) Gene Set Enrichment Analysis (GSEA) of CDK14 associated with several cell proliferation-related signaling pathways, including DNA replication (p-value=0.008) in A, mismatch repair (p-value=0.033) in B, and P53 signaling pathway in C (p-value=0.008), in ovarian cancer cell lines from CCLE. (D) Detection of SK3R-PTX cell viability after si-CDK14 transfection by the CCK-8 assay. (E) Cell cycle detection in SK3R-PTX cells after CDK14-siRNA (si-CDK14) transfection by flow cytometry. The histogram shows the percentage of cell population in each phase. (F) Detection of OV3R- PTX cell viability after si-CDK14 transfection by the CCK-8 assay. (G) Cell cycle detection in OV3R-PTX cells after si-CDK14 transfection by flow cytometry. The histogram shows the percentage of cell population in each phase. *, P < 0.05; **, P < 0.01.

Gene set enrichment analysis (GSEA) showed that CDK14 was associated with several cell proliferation-related signaling pathways, including DNA replication (p-value = 0.008) (Fig. 5A), mismatch repair (p-value = 0.033) (Fig. 5B), and P53 signaling pathway (p-value = 0.008) (Fig. 5C). Knockdown of CDK14 decreased SK3R-PTX cell viability detected by the CCK-8 assay (Fig. 5D). Flow cytometry showed a significant cell cycle arrest at the G2/M phase after the knockdown of CDK14 (Fig. 5E). Similar results were also observed in OV3R-PTX cells after CDK14 knockdown (Fig. 5F and 5G), suggesting that CDK14 is involved in PTX- resistant cell proliferation.

**Figure 6:**
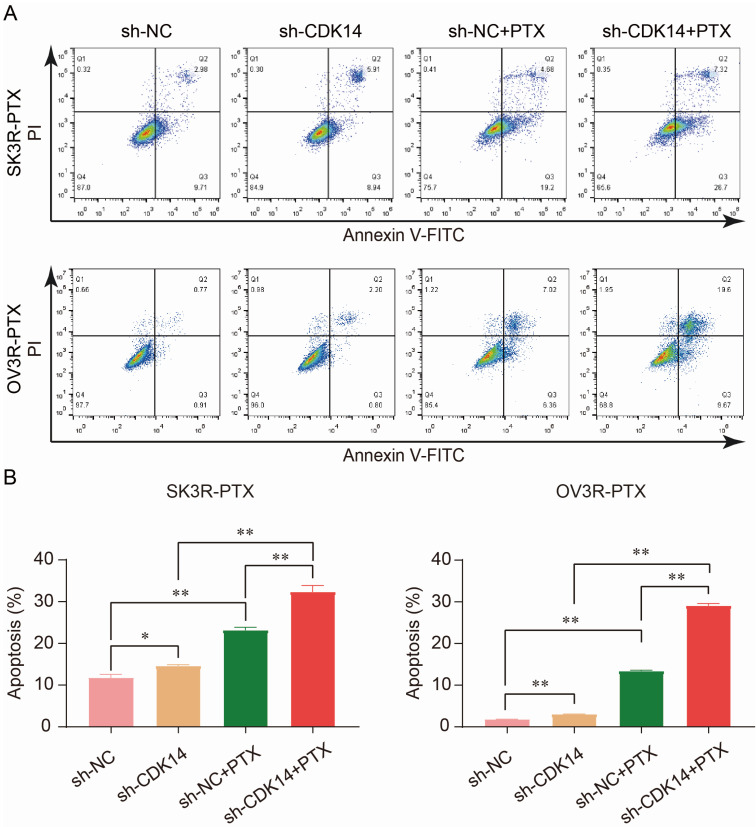
Effect of CDK14 on cell apoptosis. (A) Detection of apoptotic cells by flow cytometry. SK3R-PTX and OV3R-PTX cells were infected with CDK14-shRNA (sh-CDK14) or negative control-shRNA (sh-NC), followed by treatment of paclitaxel (PTX) for 24 h. (B) Total apoptotic cells were counted in each group. n = 3; *, P < 0.05; **, P < 0.01.

PTX is an effective anti-tumor chemotherapeutic agent which can kill cancer cells by inducing cell apoptosis. To examine the effect of CDK14 on PTX sensitivity and apoptosis, two PTX-resistant cells were stably infected with sh-CDK14 or sh-NC, followed by the treatment of an IC_50_ concentration of PTX. Flow cytometry analysis showed that the number of apoptotic cells increased after the knockdown of CDK14 by sh-CDK14 compared to sh-NC, and this result was even more significant in the presence of PTX in SK3R-PTX and OV3R-PTX cells (Fig. 6A and 6B), indicating that CDK14-shRNA is an inducer of cell apoptosis and a sensitizer of PTX.

### CDK14 is directly regulated by the TGF-β signaling pathway

**Figure 7:**
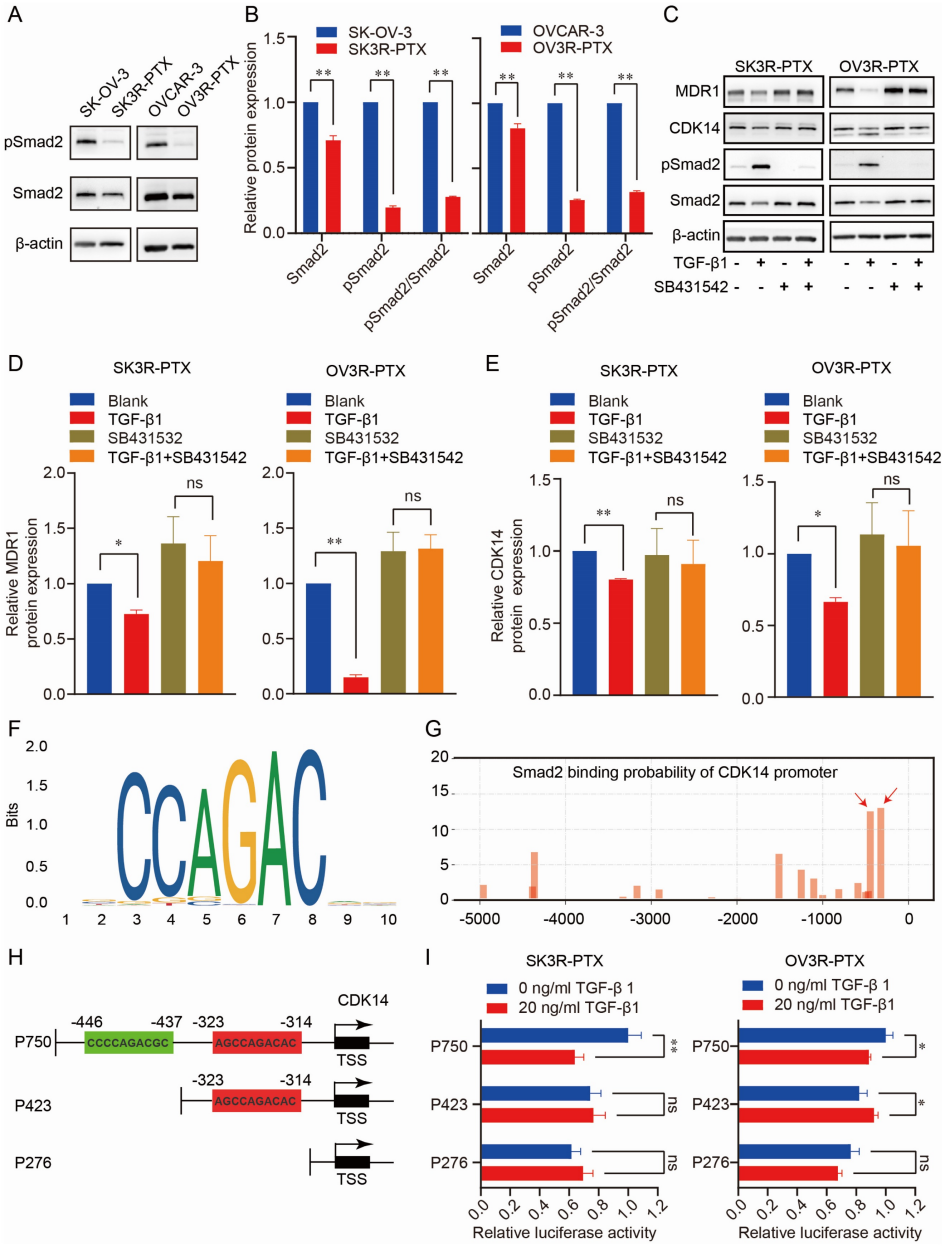
Effect of TGF-β signaling pathway on CDK14 expression in PTX-sensitive (SK-OV- 3 and OVCAR-3) and PTX-resistant (SK3R-PTX and OV3R-PTX) cells. (A, B) Detection of the expression of TGF-β signal transducer protein Smad2 and its phosphorylated Smad2 (pSmad2) in SK-OV-3, SK3R-PTX, OVCAR-3, and OV3R-PTX cells by Western blot. Histograms show the semi-quantification analysis of blotting bands by ImageJ. (C-E) Detection of MDR1, CDK14, and Smad2 proteins in PTX-resistant cells. SK3R-PTX and OV3R-PTX cells were treated with or without TGF-β (10 ng/ml) in the presence or absence of a TGF-β receptor inhibitor SB431542 (10 mM) for 48 h. The protein expression was detected by Western blot. Histograms show the quantification analysis of blotting bands. (F) The logo of the Smad2 Binding Element (SBE) motif on the promoter of CDK14 was predicted by the JASPAR database (https://jaspar.genereg.net/). (G) The binding sites of Smad2 on the promoter region - 5000 bp upstream of the CDK14 transcriptional start site (TSS) were predicted by the TFoTF analysis. Red arrows indicate the predicted Smad2 binding sites with high scores. (H) Schematic illustrations of 3 plasmid constructs. The P750 plasmid contains 750 bp with two Smad2 binding sites, the P423 plasmid contains 423 bp with one Smad2 binding site, and the P276 plasmid contains 276 bp without a Smad2 binding site located upstream of the CDK14 TSS on the CDK14 promoter. (I) Detection of luciferase activities in SK3R-PTX and OV3R- PTX cells after transfection with different CDK14 promoter-reporter constructs for 24 h in the absence or presence of TGF-β1 (0 or 20 ng/ml). After 48 h treatment of TGF-β1, the Dual Luciferase Assay was applied. Data were presented as mean ± SEM (n=3). *, P <0.05; **, P<0.01; ns, not significant.

To explore the regulatory mechanism of CDK14 expression, we analyzed the CDK14-related signaling pathways in pan-cancer from the TCGA database. GSEA analysis showed that CDK14 was closely related to the TGF-β signaling pathway in OC (Fig. S4). To test whether the TGF-β signaling was intact in PTX-sensitive and -resistant cells, the TGF-β transducer protein Smad2 was examined. Indeed, the TGF- β signaling pathway was much less active in PTX-resistant cells (SK3R-PTX and OV3R-PTX) than in the sensitive cells (SKOV-3 and OVCAR-3) as the level of phosphorylated Smad2 (pSmad2), an active transducer protein of TGF-β signaling, was lower in PTX-resistant cells (Fig. 7A, B).

Administration of 10 ng/ml TGF-β1 increased pSmad2 and decreased CDK14 and MDR1 (Fig. 7C-7E), indicating intact TGF-β signaling and responsiveness within the PTX-resistant cells. In the presence of a TGF-β receptor inhibitor SB431542, the suppressing effect of TGF-β1 on CDK14 and MDR1 expression was abolished. To prove whether Smad2 as a transcription factor directly binds to the promoter of CDK14 to regulate CDK14 expression, we analyzed the Smad Binding Element (SBE) on the promoter of the *CDK14* gene using the JASPAR database (https://jaspar.genereg.net/) and a logo of SBE was shown (Fig. 7F). Next, we predicted the possible binding sites of the SBE with different scores in the region -5000 bp upstream of the CDK14 transcription start site (TSS) using our house-made transcription factor prediction tool TFoTF 30. Two binding sites with high scores (more than 10) according to the TFoTF algorithm were selected for further experimental validations (Fig. 7G). Three luciferase reporter plasmids (named P750, P423, and P276) containing different lengths of sequences upstream of CDK14 TSS were constructed. The P750 plasmid as a wild-type control included 750 bp upstream of CDK14 TSS with two high-scored Smad2 binding sites. Two truncated plasmids (P423 and P276) included 423 bp with one SBE and 276 bp without SBE (Fig. 7H). Dual luciferase assays showed that relative luciferase activity was decreased in SK3R-PTX and OV3R-PTX cells transfected with the P750 plasmids, but not with P423 or P276 plasmids, after treatment with 10 ng/ml (less effective, data not shown) and 20 ng/ml of TGF-β1 (Fig. 7I). These data indicate that Smad2 directly binds to the region -437 to -446 upstream of CDK14 TSS to downregulate the expression of CDK14.

## Discussion

The present study reveals the expression and function of CDK14 in OC and the association of CDK14 with PTX resistance. Overexpression of CDK14 was found in OC tissues and was related to the poor prognosis of patients. Furthermore, CDK14 expression was high in PTX-resistant cells. The knockdown of CDK14 sensitized OC cells to PTX. Finally, the regulatory mechanisms of CDK14 on PTX resistance were explored in two models of OC cells.

CDK14 is a member of cyclin dependent kinases family involved in cell cycle progression and cell proliferation 14, 31. CDK14 is overexpressed in many cancers and the dysregulation of CDK14 in OC has been reported 32. It has been shown that CDK14 overexpression is associated with poor prognosis in patients with esophageal squamous cell carcinoma and non-small cell lung cancer 17, 33. Here, we demonstrate that the overexpression of CDK14 was also related to poor prognosis in patients with OC. Remarkably, to the best of our knowledge, our study shows for the first time that CDK14 expression was higher in the PTX-resistant cells than in PTX-sensitive cells, indicating that CDK14 may serve as a PTX-resistant marker for OC patients. Chemotherapy is the main adjuvant treatment for advanced OC. However, patients at advanced stages often occur resistance after the therapeutic regimens 34. Although it has been reported that the high expression of CDK14 correlates with chemoresistance in esophageal squamous cell carcinoma 17, the relationship between CDK14 and PTX resistance in OC has not been disclosed yet. Our present work shows that CDK14 overexpression correlates with PTX resistance. Furthermore, the overexpression of CDK14 was associated with the upregulation of MDR1.

Chemoresistant transporter MDR1 is a well-characterized membrane protein that acts as a drug efflux pump to flow out chemotherapy drugs, promoting cancer cell chemoresistance 23, 35. It has been reported that MDR1 is abundant and induced by chemotherapy drugs in PTX-resistant OC cells 36; and it is associated with poor prognosis in OC 37, 38. The present study shows that CDK14 expression was positively correlated with MDR1 expression by analyzing the data of 376 patients from TCGA-OV and the correlation between CDK14 and MDR1 was confirmed in the PTX-resistant cells used in this work. Knockdown of CDK14 significantly decreased MDR1 expression at mRNA and protein levels in SK3R-PTX and OV3R-PTX cells. It has also been reported that MDR1 is regulated by the Wnt/β-catenin signaling pathway and β-catenin can bind to the MDR1 promoter to activate the MDR1 transcription 39. Moreover, the CDK14/cyclin Y complex promotes Wnt signaling through phosphorylation of LRP6, a co- receptor of Wnt ligands mediating Wnt/β-catenin signaling 40, 41. Here, we observed that CDK14 downregulates β-catenin expression mainly in the nucleus of PTX-resistant cells, suggesting that the inhibition of β-catenin by CDK14-shRNA attenuates the regulation of MDR1 by the canonical Wnt/β-catenin signaling pathway and β-catenin is a key regulatory hub between CDK14 and MDR1.

Furthermore, we observed that the knockdown of CDK14 resensitized PTX-resistant OC cells to PTX and resulted in a decrease in cell proliferation and an increase in cell apoptosis. CDK14 knockdown arrested the cell cycle at the G2/M phase, which confirms the results observed in previous works in which CDK14 formed a complex with cyclin Y to arrest the cell cycle at the G2/M phase 42. However, the mechanisms underlying CDK14 regulation were unclear. Our GSEA analysis in pan-cancer from the TCGA database showed that CDK14 expression was correlated with the TGF-β signaling pathway in OC. Indeed, we found that TGF-β1 inhibited CDK14 and MDR1 expression in PTX-resistant cells, and this inhibitory effect was abolished in the presence of the TGF-β receptor inhibitor SB431542, indicating that the TGF-β signaling pathway plays a central role in CDK14-mediated PTX resistance. The new data support previous works reporting that CDKs are downstream targets in the TGF-β signaling pathway that control cell growth and division 43. A cross-talk between TGF-β and Wnt/β-catenin pathways has also been reported 44. TGF-β stimulates canonical Wnt signaling in a p38-dependent manner by decreasing the expression of the Wnt antagonist Dickkopf-1 45. Co-activation of the TGF-β and Wnt signaling pathways results in increased β-catenin and pGSK3β levels, leading to the inhibition of EMT in OC cells 46. All this evidence may indicate the existence of TGF-β/CDK14/β-catenin/MDR1 regulatory mechanisms in the development of PTX resistance.

## Conclusion

**Figure 8:**
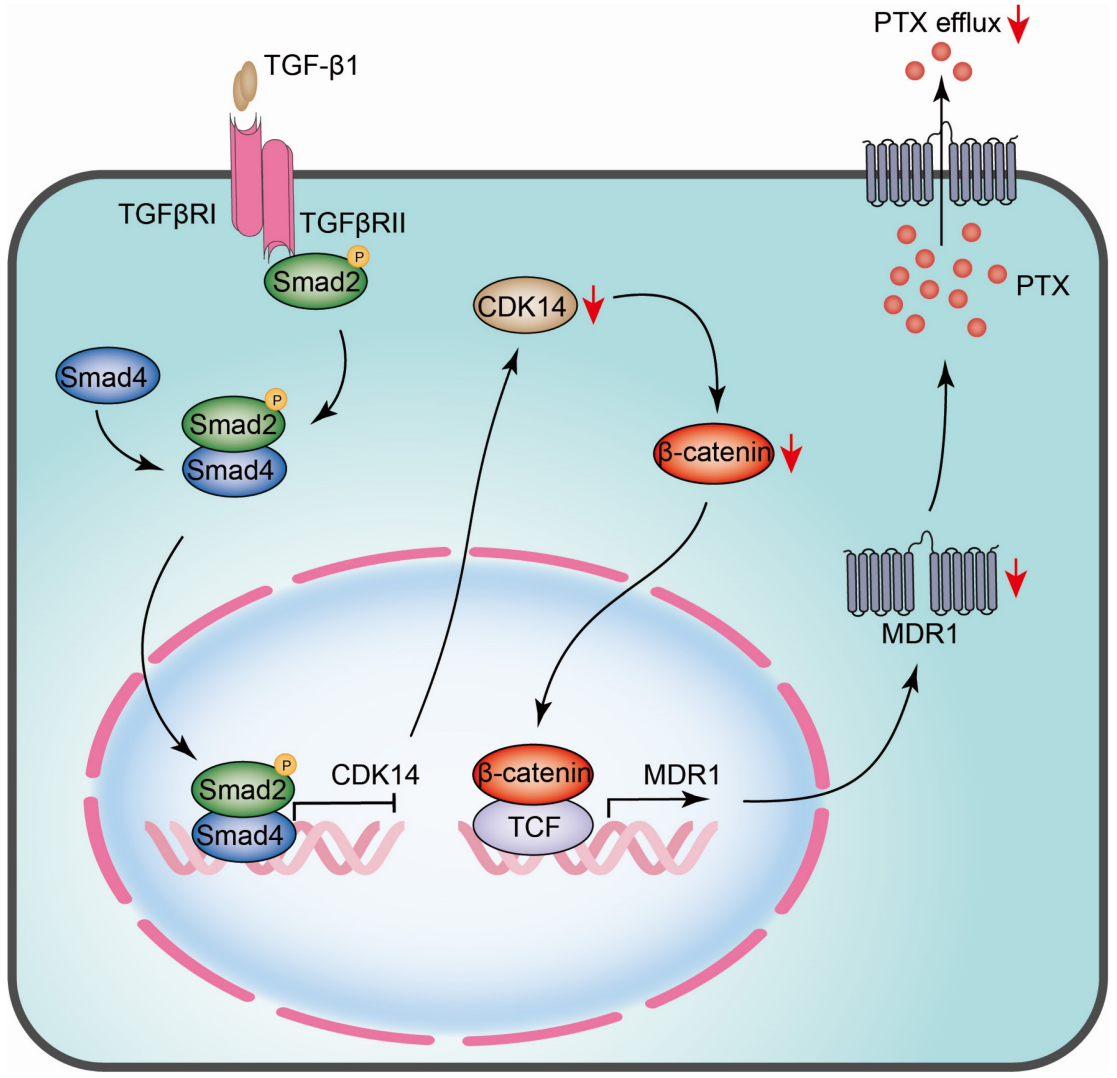
The schematic model illustrates the regulatory mechanism of CDK14 on reversing paclitaxel resistance in ovarian cancer cells. TGF-β1 phosphorylates and activates intracellular transducer protein Smad2 through TGF-β receptors (TGFβRI/II). Activated Smad2 forms complexes with Smad4, which then translocate into the nucleus and serve as transcription factors to bind to the SBE site on the promoter of CDK14, thereby inhibiting CDK14 expression. Downregulation of CDK14 protein leads to a decrease in β-catenin, whereby inhibits target gene MDR1 via β-catenin/TCF transcription factor complex. Decreased MDR1 results in reducing PTX efflux and reversing drug resistance. Red arrow, downregulation/decrease; CDK14, cyclin-dependent kinase 14; MDR1, multidrug resistance 1; SBE, Samd Binding Element; Smad, Sma and mothers against decapentaplegic; TCF, T-cell factor; TGF-β, Transforming growth factor-β.

The current study revealed that CDK14 is overexpressed in OC tissues and PTX-resistant OC cells and can serve as a PTX-resistant marker. A high level of CDK14 is correlated with poor prognosis in OC patients. CDK14 is negatively regulated by the TGF-β signaling pathway and affects MDR1 expression via the Wnt/β-catenin signaling pathway (Fig. 8). Knockdown of CDK14 inhibits cell proliferation and induces cell apoptosis, and ultimately sensitizes PTX-resistant OC cells to PTX. Thus, CDK14 is a promising therapeutic target for reversing the PTX resistance in OC.

## Supplementary Material

Supplementary figures and tables.

## Abbreviations

CDK14: cyclin-dependent kinase 14
MDR1: multidrug resistance 1
OC: ovarian cancer
PTX: paclitaxel
SBE: Smad binding element
Smad: Sma and mothers against decapentaplegic
TGF-β: transforming growth factor-β
TSS: transcription start site
Wnt: wingless-related integration site
