# Supplementary material for: Cyclin dependent kinase 14 as a paclitaxel-resistant marker regulated by the TGF-β signaling pathway in human ovarian cancer
Source: J Cancer. 2023 Aug 15;14(13):2538–51. doi: 10.7150/jca.86842 (PMC10475357; doi:10.7150/jca.86842)
Supplement: Supplementary file 1 — Supplementary figures and tables. [file jcav14p2538s1.pdf]

Supplementary file

Cyclin dependent kinase 14 as a paclitaxel-resistant marker regulated by the TGF-β signaling pathway in human ovarian cancer

Wencai Guan[1]*, Jia Yuan[1,2]*, Xin Li[1,2,], Xuzhu Gao[1], Fanchen Wang[1,2,], Huiqiang Liu[1,2], Jimin Shi[1], Guoxiong Xu[1,2]✉

[1]Research Center for Clinical Medicine, Jinshan Hospital, Fudan University, Shanghai 201508, China; [2]Department of Oncology, Shanghai Medical College, Fudan University, Shanghai 200032, China;

*These authors share first authorship

✉Corresponding author:

Guoxiong Xu, MD, PhD

Scientist, Professor of Oncology

Research Center for Clinical Medicine, Jinshan Hospital, Fudan University

1508 Longhang Road, Shanghai 201508, P.R. China

Tel: +86-21-34189990; Fax: +86-21-57039502

Email: guoxiong_xu@shmu.edu.cn

ORCID iD: 0000-0002-9074-8754

Supplementary Table S1. Sequences of shRNA, siRNA, and primer

Name	Sequence (5' → 3')	Target position
**sh-NC**		
Sense	ccggTTCTCCGAACGTGTCACGTCTCGA GACGTGACACGTTCGGAGAATTTTTTg	Scramble
Antisense	aattcAAAAAATTCTCCGAACGTGTCACG TCTCGAGACGTGACACGTTCGGAGAA	
**Sh-CDK14**		
Sense	ccggGGCAAAGAGTCACCTAAAGTTCTC GAGAACTTTAGGTGACTCTTTTTg	nt 415-435
Antisense	aattcAAAAAGGCAAAGAGTCACCTAAA GTTCTCGAGAACTTTAGGTGACTCTTT GCC	
**si-NC**		
Sense	UUCUCCGAACGUGUCACGUTT	Scramble
Antisense	ACGUGACACGUUCGGAGAATT	
**si-CDK14**		
Sense	GGCAAAGAGUCACCUAAAGUUTT	nt 415-435
Antisense	AACUUUAGGUGACUCUUUGCCTT	
**CDK14 PCR primer**		
Forward	AGATGACACCACCTTTGATG	nt 409-428
Reverse	CCTCAGGAATTGTGTCCAG	nt 509-491
**MDR1 PCR primer**		
Forward	CATTTGGCAAAGCTGGAGAG	nt2792-2811
Reverse	ATCATTGGCGAGCCTGGTAG	nt2928-2909
**β-actin PCR primer**		
Forward	TCATCACCATTGGCAATGAG	nt824-843
Reverse	CACTGTGTTGGCGTACAGGT	nt978-959

The position in the CDK14 mRNA sequence (GenBank Accession: NM_001287135), MDR1 mRNA sequence (GenBank Accession: NM_000927), and β-actin mRNA sequence (GenBank Accession: NM_001101) is shown. The low case indicates a linker. CDK14, cyclin dependent kinase; MDR1, multidrug resistance 1; NC, negative control; nt, nucleotide; PCR, polymerase chain reaction; shRNA, short hairpin RNA; siRNA, small interference RNA.

Supplementary Table S2. Correlation between clinicopathological features and CDK14 expression in ovarian cancer.

Characteristics	CDK14 expression		P value
	High	Low	
N	9	9	
Age, mean ± SD	60.222 ± 10.604	57.667 ± 10.724	0.618 [a]
T.stage, n (%)			*0.026* [b]
T1	2 (11.1%)	7 (38.9%)	
T2	1 (5.6%)	1 (5.6%)	
T3	6 (33.3%)	1 (5.6%)	
N.stage, n (%)			0.347 [b]
N1	6 (33.3%)	3 (16.7%)	
N0	3 (16.7%)	6 (33.3%)	
M.stage, n (%)			0.153 [b]
M1	6 (33.3%)	2 (11.1%)	
M0	3 (16.7%)	7 (38.9%)	
Pathologic stage, n (%)			0.131 [b]
stage I	1 (5.6%)	5 (27.8%)	
stage II	1 (5.6%)	0 (0%)	
stage III	7 (38.9%)	4 (22.2%)	
CA125 (U/mL), median (IQR)	602.3 (101.3, 2925.1)	116 (98.9, 181.7)	0.596 [c]
CA153 (U/mL), median (IQR)	16.1 (8.28, 68.2)	11.7 (9.6, 117.2)	0.724 [c]
Histology, n (%)			0.772 [b]
HGSC	7 (38.9%)	5 (27.8%)	
CCC	1 (5.6%)	1 (5.6%)	
EC	1 (5.6%)	3 (16.7%)	

T, primary tumor; N, lymph node; M, metastasis; CA125, cancer antigen 125; CA153, cancer antigen 153; IQR, interquartile range; HGSC, high-grade serous carcinoma; CCC, clear cell carcinoma; EC, endometrioid carcinoma; n, number of cases; SD, standard deviation. Statistical test: a, Student's *t*-test; b, Fisher test; c, Wilcoxon test.

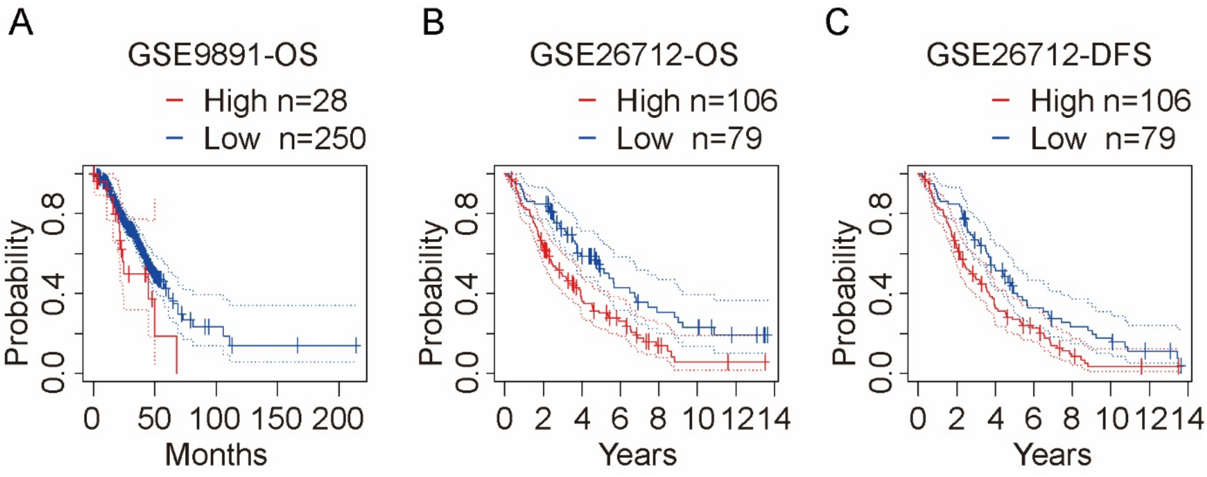

Supplementary Figure S1. Prognostic value of CDK14 in patients with ovarian cancer. (A) A comparison of overall survival (OS) between high-expression and low-expression of CDK14 was shown. Data were obtained from the GSE dataset GSE9891. (B,C) A comparison of OS and disease-free survival (DFS) between high-expression and low-expression of CDK14 was shown. Data were obtained from the GSE dataset GSE26712.

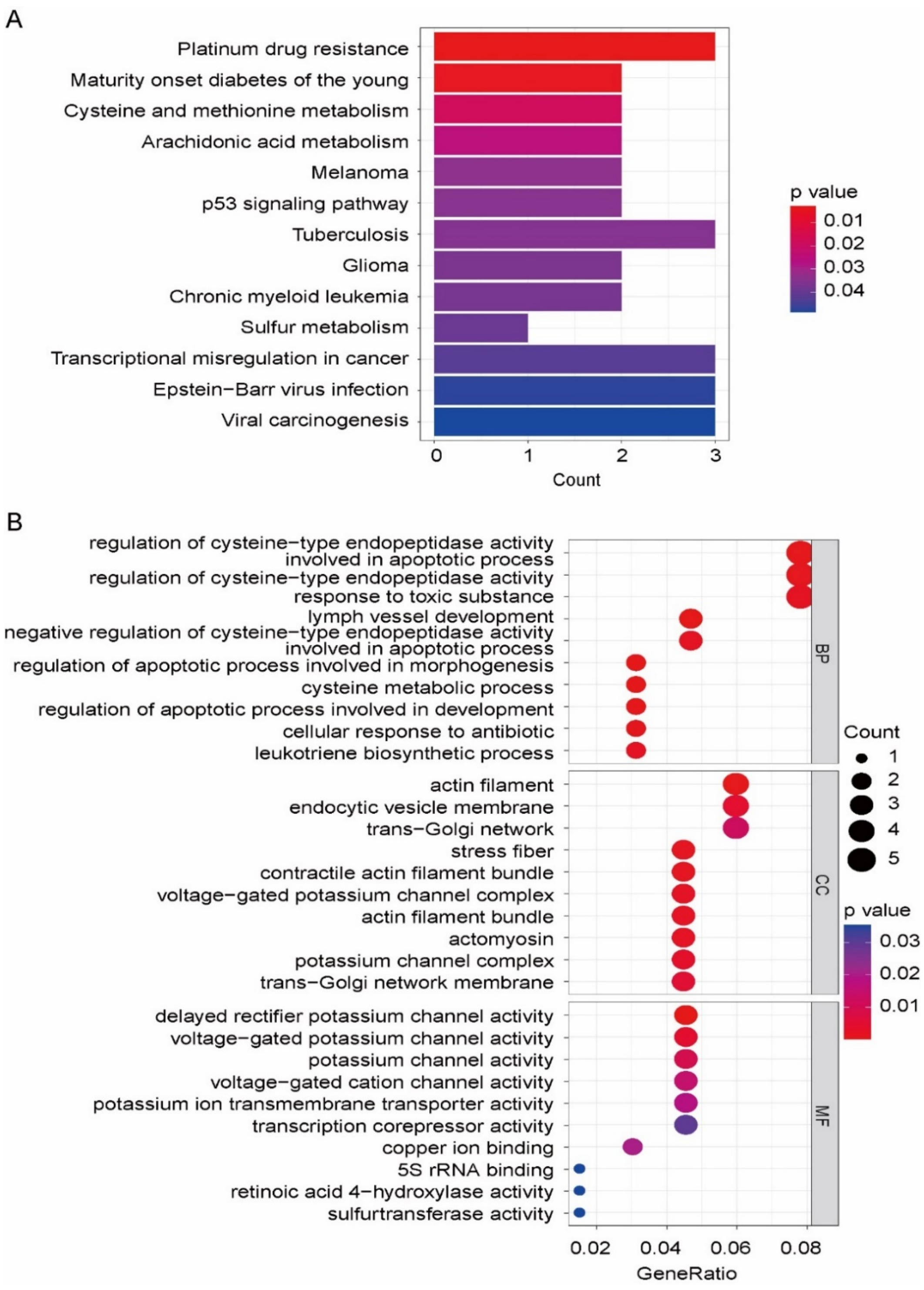

Supplementary Figure S2. Analysis of CDK14-correlated gene expression. (A) CDK14-correlated (positively or negatively) gene pathways were analyzed by KEGG pathway enrichment analysis. (B) The bubble plot showed the enrichment of CDK14-correlated genes in functional analyses, including biological process (BP), cellular component (CC), and molecular function (MF), by GO term analyses.

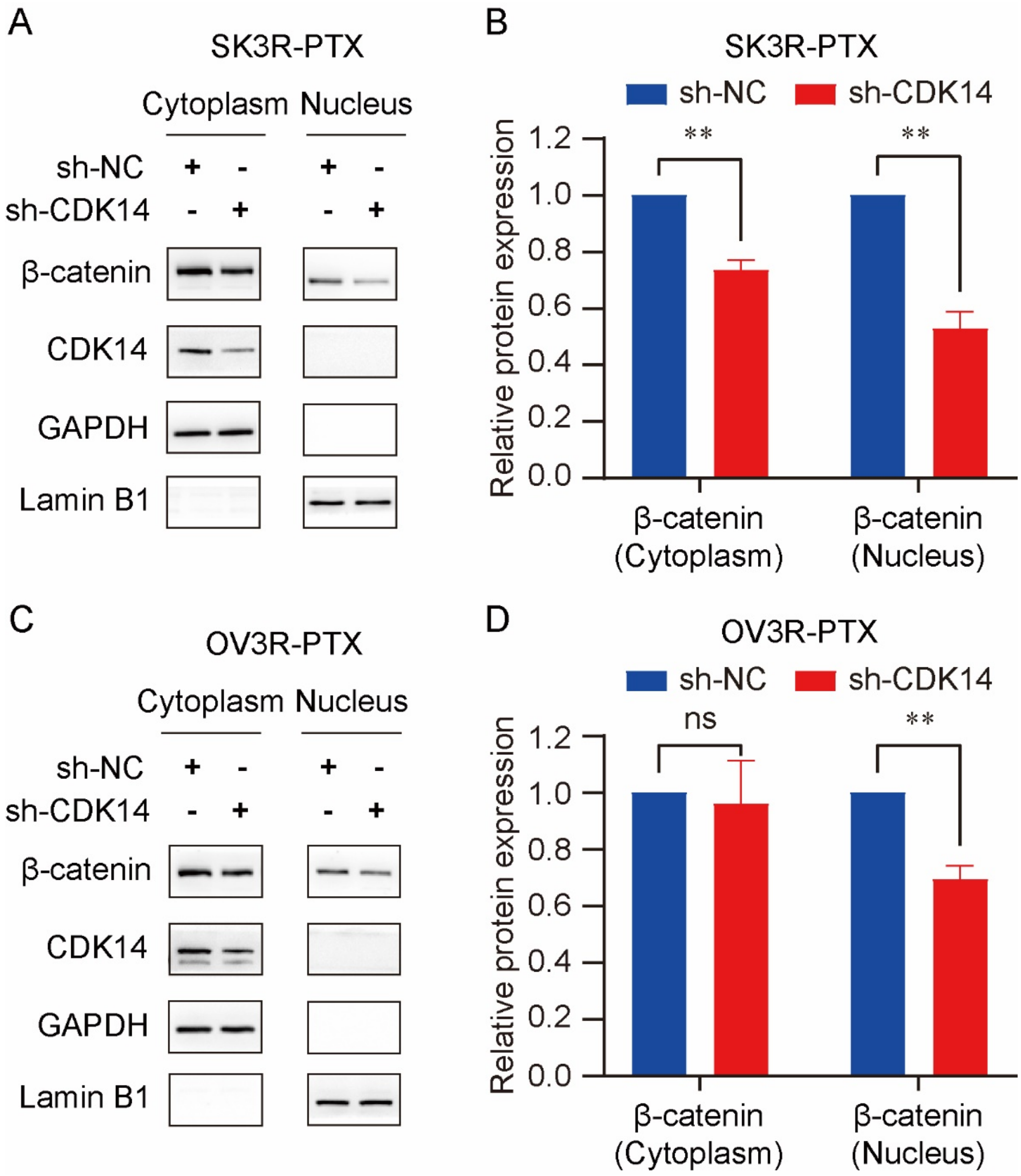

Supplementary Figure S3. Effect of CDK14-shRNA on β-catenin expression. (A) Detection of cytoplasmic and nuclear β-catenin protein expression by Western blot in SK3R-PTX cells infected with negative control-shRNA (sh-NC) and CDK14-shRNA (sh-CDK14) viruses. (B) Semi-quantitative analysis of the relative optical density of β-catenin bands in A. (C) Detection of cytoplasmic and nuclear β-catenin protein expression by Western blot in OV3R-PTX cells infected sh-NC and sh-CDK14. (D) Semi-quantitative analysis of the relative optical density of β-catenin bands in C. GAPDH and Lamin B1 were used as loading controls for the cytoplasmic and nuclear proteins. n = 3; **, $P < 0.01$; ns, no significant differences.

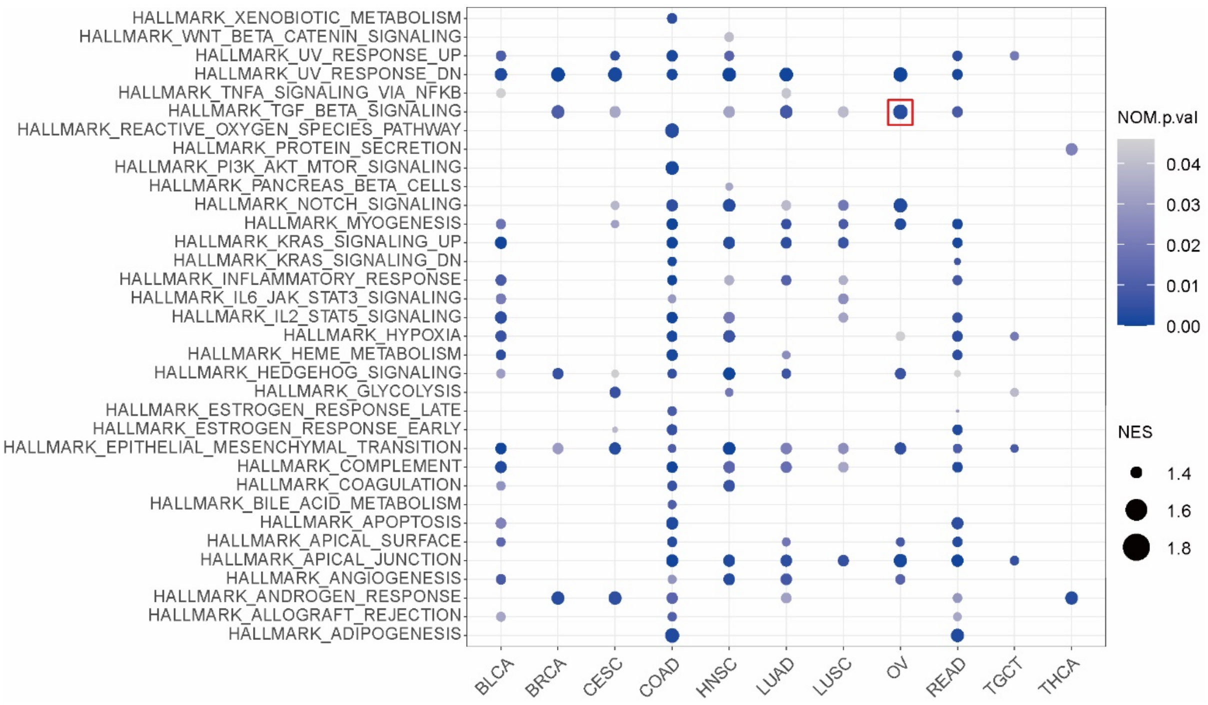

Supplementary Figure S4. CDK14-related signaling pathways in pan-cancer. Data were extracted from the TCGA database. The correlation diagram showed enriched pathways in the CDK14 high-expression group analyzed by GSEA. The red square indicates the TGF-β signaling pathway in ovarian cancer (OV).
